# A machine learning method for the identification and characterization of novel COVID-19 drug targets

**DOI:** 10.1038/s41598-023-34287-5

**Published:** 2023-05-03

**Authors:** Bruce Schultz, Lauren Nicole DeLong, Aliaksandr Masny, Manuel Lentzen, Tamara Raschka, David van Dijk, Andrea Zaliani, COPERIMOplus, Anne Funck Hansen, Sabine, Kugler Stefan Rüping, Jan Burmeister, Jörn Kohlhammer, George Sarau, Silke Christiansen, Aimo Kannt, Andrea Zaliani, Ann Christina Foldenauer, Carsten Claussen, Eduard Resch, Kevin Frank, Phil Gribbon, Maria Kuzikov, Oliver Keminer, Hendrik Laue, Horst Hahn, Jochen Hirsch, Marco Wischnewski, Matthias Günther, Saulius Archipovas, Alpha Tom Kodamullil, Andre Gemünd, Bruce Schultz, Carina Steinborn, Christian Ebeling, Daniel Domingo Fernández, Helena Hermanowski, Holger Fröhlich, Jürgen Klein, Manuel Lentzen, Marc Jacobs, Martin Hofmann-Apitius, Meike Knieps, Michael Krapp, Philipp Johannes Wendland, Philipp Wegner, Sepehr Golriz Khatami, Stephan Springstubbe, Thomas Linden, Juliane Fluck, Holger Fröhlich

**Affiliations:** 1 Department of Bioinformatics, Fraunhofer Institute for Algorithms and Scientific Computing (SCAI), 53757 Sankt, Augustin Germany; 2 Artificial Intelligence and its Applications Institute, University of Edinburgh School of Informatics, 10 Crichton St, Edinburgh, EH8 9AB UK; 3 Fraunhofer Institute for Translational Medicine and Pharmacologie (ITMP), Drug Discovery Research ScreeningPort, VolksparkLabs, Schnackenburgallee 114, 22535 Hamburg, Germany; 4 University of Bonn, Bonn-Aachen Center for IT (b-it), Friedrich Hirzebruch-Allee 6, 53115 Bonn, Germany; 5 Fraunhofer Center for Machine Learning, Sankt, Germany; 6 Center for Biomedical Data Science, Yale School of Medicine, Yale University, 333 Cedar Street, New Haven, CT 06510 USA; 7 Fraunhofer Data Protection Office, Sankt, Germany; 8 Fraunhofer IAIS, Sankt, Germany; 9 Fraunhofer IGD, Sankt, Germany; 10 Fraunhofer IKTS, Sankt, Germany; 11 Fraunhofer ITMP, Sankt, Germany; 12 Fraunhofer MEVIS, Sankt, Germany; 13 Fraunhofer SCAI, Sankt, Germany; 14 ZB MED Information Centre for Life Sciences, Cologne, Germany

**Keywords:** Machine learning, Target identification, Drug safety

## Abstract

In addition to vaccines, the World Health Organization sees novel medications as an urgent matter to fight the ongoing COVID-19 pandemic. One possible strategy is to identify target proteins, for which a perturbation by an existing compound is likely to benefit COVID-19 patients. In order to contribute to this effort, we present GuiltyTargets-COVID-19 (https://guiltytargets-covid.eu/), a machine learning supported web tool to identify novel candidate drug targets. Using six bulk and three single cell RNA-Seq datasets, together with a lung tissue specific protein-protein interaction network, we demonstrate that GuiltyTargets-COVID-19 is capable of (i) prioritizing meaningful target candidates and assessing their druggability, (ii) unraveling their linkage to known disease mechanisms, (iii) mapping ligands from the ChEMBL database to the identified targets, and (iv) pointing out potential side effects in the case that the mapped ligands correspond to approved drugs. Our example analyses identified 4 potential drug targets from the datasets: AKT3 from both the bulk and single cell RNA-Seq data as well as AKT2, MLKL, and MAPK11 in the single cell experiments. Altogether, we believe that our web tool will facilitate future target identification and drug development for COVID-19, notably in a cell type and tissue specific manner.

## Introduction

The ongoing COVID-19 pandemic led to millions of deaths and huge economic costs worldwide. While effective vaccinations are now widely available in developed countries, there are still a considerable number of infected people worldwide^1^. As of December 2022, there were a total of 12 treatment options that have either been authorized by the European Union (EU), approved by the United States Food and Drug Administration (USFDA), or are being utilized under an emergency use authorization (EUA) in the United States (U.S.) for treating severe cases of COVID-19^2,3^. These options include anakinra (binds to the IL-1 receptor; EU & EUA), baricitinib (a JAK inhibitor; US FDA), ritonavir (a virus protease inhibitor; EU & EUA), tocilizumab (an IL-6 antibody; EU & EUA), remdesivir (a viral RNA polymerase inhibitor; EU & US FDA), molnupiravir (a ribonucleoside that introduces errors during viral replication; EUA), nirmatrelvir/ritonavir (virus protease inhibitors; EUA), as well as several monoclonal antibodies against the spike protein including regdanvimab (EU), bebtelovimab (EUA), sotrovimab (EU), tixagevimab/cilgavimab (EU & EUA) and casirivimab/imdevimab (EU). Though casirivimab/imdevimab, bamlanivimab/etesevimab and sotrovimab were previously used in the U.S. under the EUA, they are currently no longer authorized due to the rise of SARS-CoV-2 variants in this region which are resistant to these antibodies. Due to this limited number of available compounds, there is clearly an unmet need for new, highly effective and well tolerated drugs which can be administered to prevent progression to a severe disease stage.

Given the pressing need for effective novel treatments and that traditional drug development requires a massive time investment, there has been a growing interest recently in utilizing drug repositioning for COVID-19^4,5^. To date, there are three general strategies being applied in the field of COVID-19 drug repositioning^6^:

1. *Same Target—New Virus*: This strategy focuses on the idea of reusing an approved antiviral drug with a known target on a new virus. An example of this strategy is remdesivir, which was originally developed against Ebola^7^.
2. *Same Target - New Indication*: This strategy refers to using a drug known to modulate an essential pathway in human cells during infection to treat a disease that affects said pathway. One example is tocilizumab, an IL6 antibody that was originally approved for treating cytokine release syndrome, being administered to patients to inhibit the pro-inflammatory pathways that are activated in severe COVID-19 infections^8^.
3. *New Target - New Indication*: This strategy focuses on using existing compounds against novel targets, which are essential during viral infection^9–11^.

Existing computational approaches focus on integrating established biological knowledge from recent literature^12^ in order to predict novel targets of existing drugs^13^, identify targets for which a perturbation from a known compound would likely affect the virus-host interaction^14^, or modify the response of infected cells^15^. In addition to these methods, several machine learning techniques have also been applied to this task including link prediction within a human interactome^16^ as well as methods combining knowledge graphs with gene expression profiles^14,17^, typically using a single gene expression dataset. To our knowledge, there has been no attempt so far to perform a more robust identification of viable targets based on a wider range of bulk and single cell RNA-Seq (scRNA-Seq) datasets, nor is there a suitable tool available to the scientific community which supports such an activity. Ideally, such a tool should—beyond predicting candidate targets - address the additional considerations of target identification including its degree of disease linkage, any associated target-related safety issues, and its technical feasibility such as its druggability^18^.

In the work presented here, we sought to fill this gap by developing a web-tool, GuiltyTargets-COVID-19 (https://guiltytargets-covid.eu/) that can

1. Make use of machine learning to prioritize candidate targets in a tissue specific manner and assess their druggability.
2. Unravel their linkage to known disease-associated human proteins and virus–host interactions.
3. Map them to additional ligands derived from the ChEMBL database.
4. Identify any potential safety issues.

We demonstrate the utility of our web tool by applying it to six bulk and three single cell RNA-Seq datasets.

## Results

### GuiltyTargets-COVID-19 web tool

We start by providing a high level overview about the capabilities of the GuiltyTargets-COVID-19 web tool. The web application initially allows the user to browse through a ranked list of potential targets generated using six bulk RNA-Seq and three single cell RNA-Seq datasets applied to a lung specific protein–protein interaction (PPI) network reconstruction. Our website is also equipped with several filtering options to allow the user to quickly obtain the most relevant results. The candidate targets were ranked using a machine learning algorithm, GuiltyTargets^19^, which aims to quantify the degree of similarity of a candidate target to other known (candidate) drug targets. Further details about GuiltyTargets are outlined in the Methods section of this paper.

**Figure 1:**
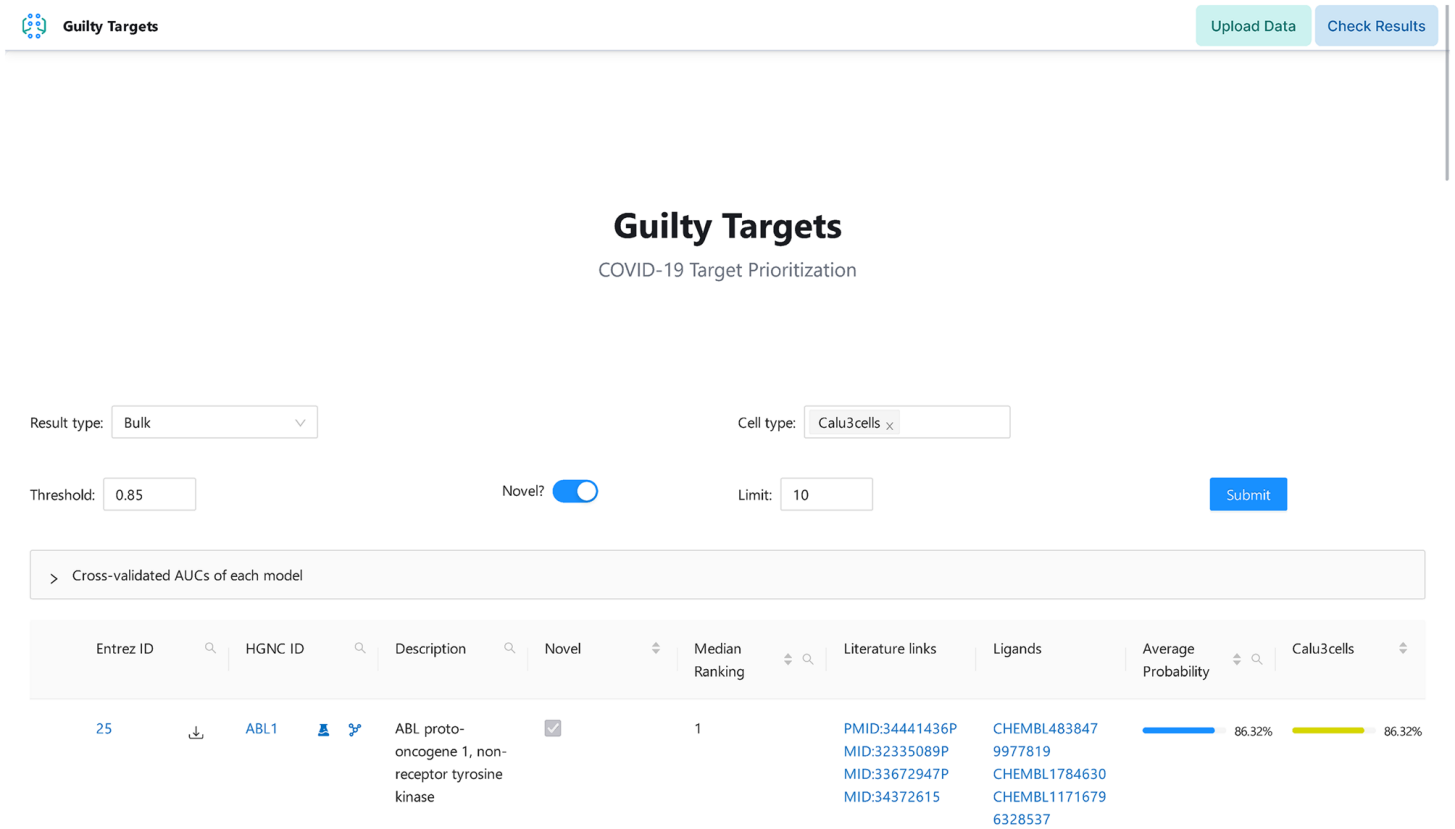
Screenshot of the GuiltyTargets-COVID-19 web application available at https://guiltytargets-covid.eu/.

The user can retrieve a consensus ranking of any combination of datasets desired (Fig. 1). For each protein listed, its level of differential gene expression (upregulated, downregulated, no differential expressed) is displayed using a color coding system in addition to its association with COVID-19 as described in the literature. This latter feature is accomplished using an automated web search of scientific articles from PubMed that mention the protein in combination with COVID-19.

Though we provide nine different RNA-Seq datasets to explore, our tool also allows one to upload their own gene expression data. Uploaded data is sent through the GuiltyTargets algorithm and, after a short period of time, a ranking of candidate proteins is made available to the user to download and explore.

**Figure 2:**
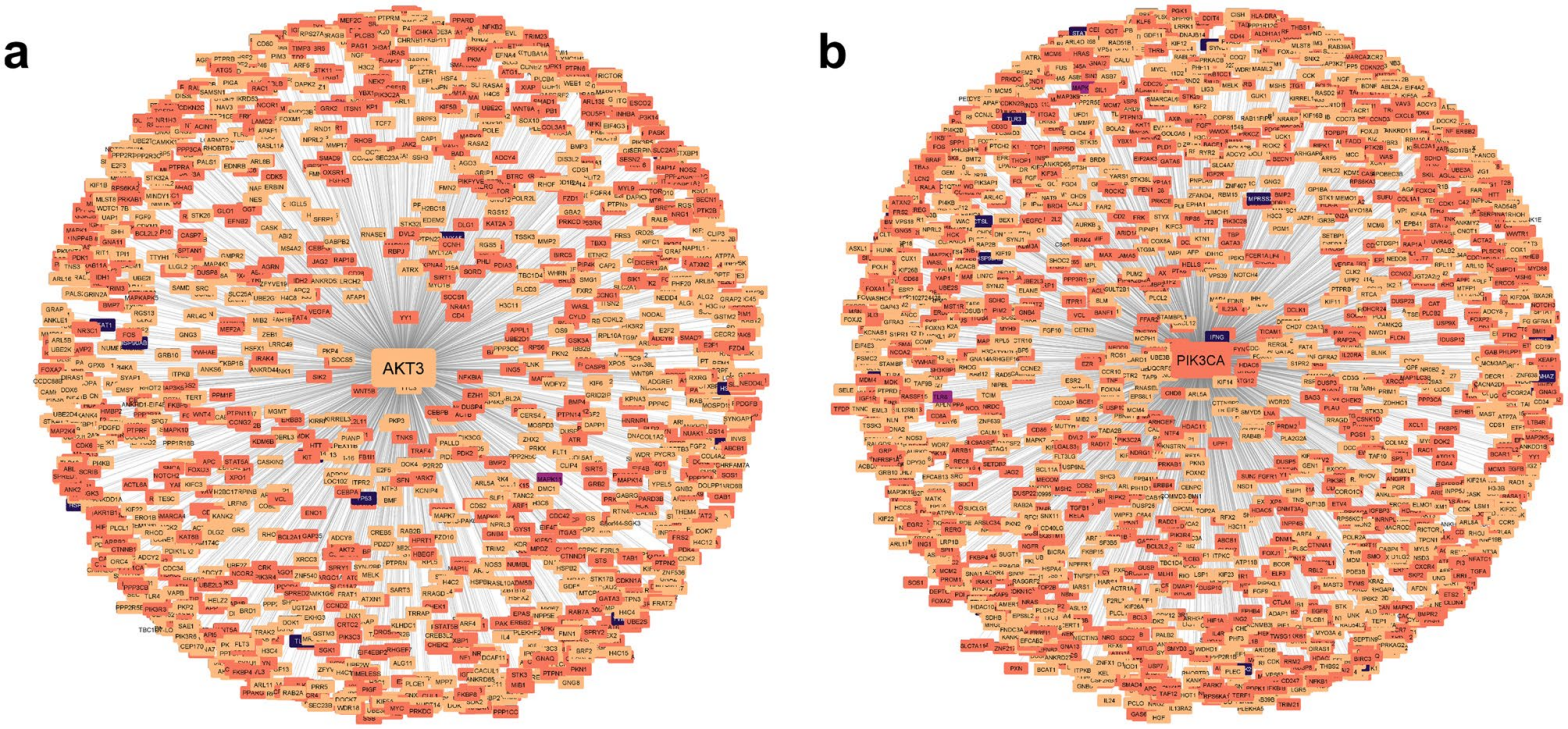
First degree neighbors of the (**a**) AKT3 and (**b**) PIK3CA proteins. Nodes are colored according to their associations: light orange means no virus or human association was found, dark orange indicates only human association, purple signifies viral association, and and dark blue nodes are proteins with associations to both viral mechanisms and human processes. The neighboring proteins and their associations for AKT3 and PIK3CA are outlined in Supplementary Data S1 and S2, respectively.

To further elucidate their linkage to known disease mechanisms, GuiltyTargets-COVID-19 enables one to explore the neighborhood of any given candidate target within the lung tissue specific PPI network reconstruction (Fig. 2). The network is labeled with information about known disease associations in humans in addition to virus-host interactions.

Importantly, in order to present the user with a list of possible drug candidates for a given protein, we parsed the ChEMBL database to generate a mapping of known ligands for each of the prioritized proteins and included this information in our web application. Direct links to the ligands’ description pages were added to GuiltyTargets-COVID-19 so that researchers can quickly explore the each compound’s profile.

To point out potential target related safety issues, GuiltyTargets-COVID-19 includes a list of adverse effects for each target-linked compound, all of which were derived from the NSIDES database^20^. By making this information readily available, the user can quickly decide which compounds for a given target are most viable.

Altogether, GuiltyTargets-COVID-19 implements a comprehensive workflow involving computational target prioritization supplemented with annotations from several key databases.

### Demonstration of use

#### Ranking of candidate targets

In the following sections, we demonstrate the utility of GuiltyTargets-COVID-19 based on the analysis of 6 bulk RNA-Seq and 3 single cell RNA-Seq datasets. A detailed overview of the data and workflow can be found in the “Differential gene expression” section of the Methods. In brief, GuiltyTargets-COVID-19 maps differentially expressed genes in each of these datasets to a lung tissue specific, genome-wide PPI network, which was constructed using data from BioGRID^21^, IntAct^22^ and STRING^23^ (see “PPI Network Construction” in Methods). Users can choose a combination of these datasets and the tool will present a ranking of each protein for each selected dataset based on its similarity to known drug targets. Additionally, a consensus ranking is also calculated if multiple datasets were selected.

For our analysis, we initially performed a ranking for each individual dataset. This ranking was performed using the GuiltyTargets positive-unlabeled machine learning algorithm^19^, which combines a PPI network, a differential gene expression (DGE) dataset, and a list of included nodes that are labeled as putative targets. Based on these results, GuiltyTargets then quantifies the probability that a candidate protein could be labeled as a target as well. In order to create a usable model, GuiltyTargets-COVID-19 was trained using a set of 218 proteins targeted by small compounds extracted from ChEMBL. This set of proteins was previously found to be involved in cellular response mechanisms specific to COVID-19 that have been shown to be transcriptionally dysregulated in several bulk RNA-Seq datasets^15^. The set of 218 proteins may thus be regarded as an extendable set of candidate targets. We chose this approach as there are currently very few approved drugs for COVID-19 (7 as of December 2022 in the European Union), hence making a machine learning model based ranking with respect to only known targets of approved drugs rather questionable.

In order to maximize transparency, GuiltyTargets-COVID-19 also reports the ranking performance of the GuiltyTargets machine learning algorithm that is calculated using the cross-validated area under receiver operator characteristic curve (AUC). As show in Fig. 6, the cross-validated AUCs found for each of the nine datasets used in this work were found to be between 85% and 90%, which align with the results reported in^19^. Additional details regarding the algorithm’s performance can be found in the Methods Section.

#### Consistently top ranked targets demonstrate disease association

For our use case, we focused on proteins with a predicted target likelihood higher than 85% in each of the nine datasets. This resulted in 51–67 candidate targets for each of the bulk RNA-Seq datasets and 45–65 candidate targets for each of the scRNA-Seq datasets. By enabling the filter option “novel” in our web tool, we can select for those prioritized targets that are not among the original set of 218 proteins labeled as known targets and used for training the model.

**Figure 3:**
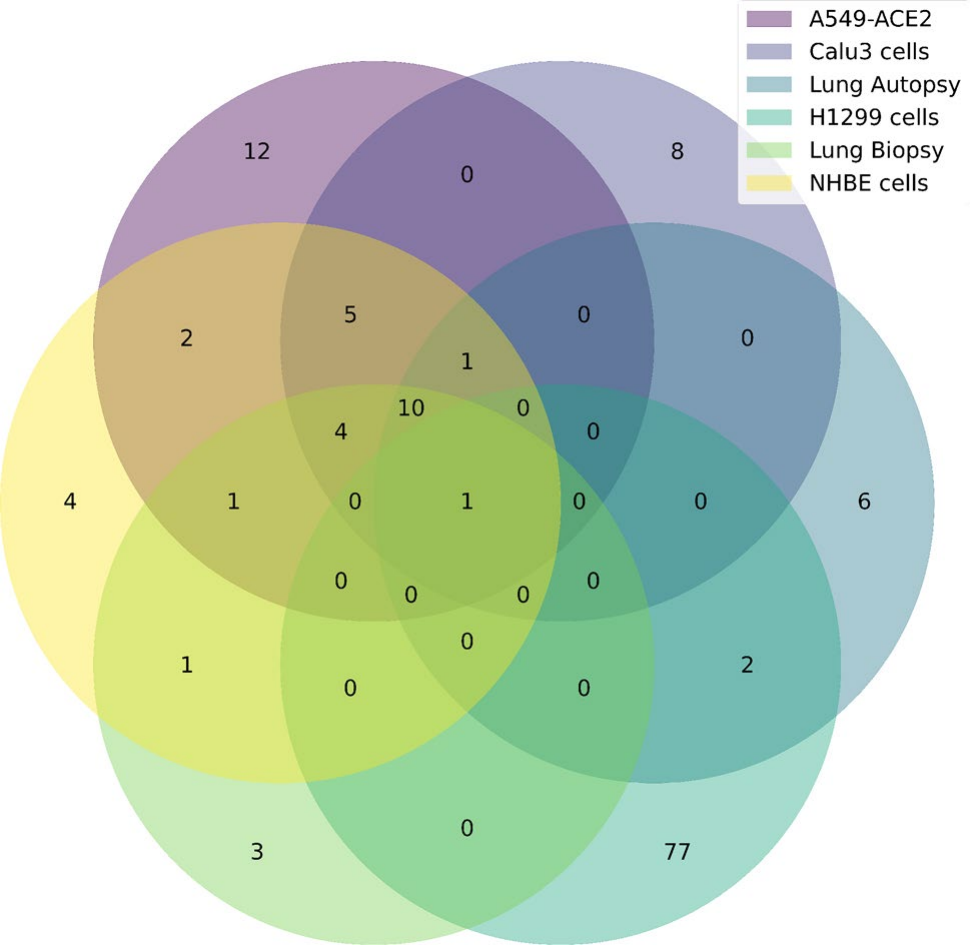
Venn diagram of the number of prioritized targets from the bulk RNA-Seq datasets.

Among these prioritized targets, there was a considerable difference between the analyzed bulk RNA-Seq data, with only a single protein target appearing among the top candidates for all 6 datasets: AKT3 (Fig. 3). AKT3 is of great interest in COVID-19 research as the PI3K/AKT signaling pathway plays a central role in cell survival. Moreover, researchers have observed an association between this pathway and coagulopathies in SARS-CoV-2 infected patients^24^. It has been suggested that the PI3K/AKT signaling pathway can be over-activated in COVID-19 patients either by direct or indirect mechanisms, thus suggesting this pathway may serve as a potential therapeutic target^25^.

To better understand the relationship of AKT3 with known COVID-19 disease mechanisms, the user can also download a CSV file comprised of the direct (first-degree) neighbors of AKT3 in the lung tissue specific PPI network used for our analysis. Each first-degree neighbor is additionally annotated to indicate whether the corresponding protein is associated with either the disease or with the virus itself. Figure 2a provides a visualization of the AKT3 neighbor network generated using Cytoscape 3.9.1^26^.

Interestingly, a larger number of shared prioritized protein targets can be found among the scRNA-Seq data. Based on the 17 cell types identified in the three datasets, four common target candidates were identified: AKT2, AKT3, MAPK11, and MLKL. The presence of AKT3, as well as its isoform AKT2, in our list of prioritized targets supports the predicted association of the PI3K/AKT signaling pathway with COVID-19 as observed in our analysis of the bulk RNA datasets. Interestingly, our analysis of the single-cell datasets revealed two additional proteins of interest, MAPK11 and MLKL. MAPK11 is targeted by the compound losmapimod, which was tested against COVID-19 in a (terminated) phase III clinical trial (NCT04511819). The trial ended in August 2021 “due to the rapidly evolving environment for the treatment of Covid-19 and ongoing challenges to identify and enroll qualified patients to participate” (https://clinicaltrials.gov/ct2/show/NCT04511819). MLKL is a pseudokinase that plays a key role in TNF-induced necroptosis, a programmed cell death process. Recent evidence suggests that it can become dysregulated by the inflammatory response due to SARS-CoV-2 infection^27^. According to the DGldb database^28^ (which is cross-referenced by GuiltyTargets-COVID-19), the protein is also druggable and thus may serve as a therapeutic target.

Overall, these results demonstrate that GuiltyTargets-COVID-19 has the capability of identifying candidate targets with a clear disease association as well as assessing their potential druggability.

#### Cell type specific target prioritization

After analyzing the top ranked protein targets shared by each group of RNA-Seq data, we next sought to characterize those candidates found in unique cell types (Table 1). Interestingly, we found that PIK3CA was only ranked among the top therapeutic candidates in goblet cells. Goblet cells are modified epithelial cells that secrete mucus on the surface of mucous membranes of organs, particularly those of the lower digestive tract and airways. Dactolisib is a compound targeting PIK3CA that has been tested in a phase II clinical trial for its ability to reduce COVID-19 disease severity (NCT04409327). The trial was terminated due to an insufficient accrual rate (https://clinicaltrials.gov/ct2/show/NCT04409327). Figure 2b depicts the PIK3CA protein and its first-degree neighbors as defined by the PPI network used in the GuiltyTargets-COVID-19 algorithm.

Another interesting drug we identified during our analysis is the compound varespladib, a compound that is currently being tested in a phase II clinical trial (NCT04969991) and which targets PLA2G2A, a potential protein target that primarily affects NKT cells (Table 1). To better support the user in finding more information about the disease context of such candidate targets, GuiltyTargets-COVID-19 also includes links to PubMed articles in which the protein and its roles in COVID-19 are discussed. Identification of relevant articles is discussed in the the “Methods” section.

Altogether, these results demonstrate that the tool presented here can be used for cell type specific target prioritization as well as aiding in characterizing the proteins in the context of COVID-19.

**Table 1:** Candidate targets solely prioritized within one cell type, but not in others.

Cell type	Unique candidate targets
Basal cells	–
B cells	GNAI1
Ciliated cells	RPL19
Club cells	EPHB2
Dendritic cells	RIPK3, RPS14
Epithelial cells	–
Goblet cells	PIK3CA
Ionocytes	CRY1, PIM3, ACVRL1, CDC42BPA
Macrophages	IP6K2, GHRL
Mast cells	RIPK1, CDK17
Neuro cells	HSF1, MST1R
NKT cells	PLA2G2A, PRKAB1, ABCG2
NK cells	PDCD4
Secretory cells	GRK2
Squamous	CYP1A1, GNAI3
Tuft cells	CDK5, PASK
T cells	HCK

#### Identifying active ligands

GuiltyTargets-COVID-19 also includes a feature for identifying small compound ligands from the ChEMBL database with reported activity (pChEMBL > 5) against candidate targets. In our use case, we were able to identify 186 ligands for AKT3, the top prioritized target across bulk RNA-Seq datasets. Furthermore, 126 ligands were mapped to the four candidate targets that were found among all single cell RNA-Seq datasets. A complete report of the number of ligands mapped to protein targets unique for a given cell type can be found in Table 2. We observed a high imbalance of mapped ligands for different cell types with secretory cells being targeted by the vast majority of compounds.

In total, these results demonstrate the ability of GuiltyTargets-COVID-19 to efficiently identify active ligands against candidate targets, thus supporting researchers in rapidly identifying potential new drugs for therapeutic intervention or repurposing.

**Table 2:** The number of active ligands mapped to cell type specific, highly prioritized protein targets.

Cell type	Number of mapped ligands
Basal cells	–
B cells	–
Ciliated cells	–
Club cells	3
Dendritic cells	–
Epithelial cells	–
Goblet cells	2
Ionocytes	3
Macrophages	10
Mast cells	2
Neuro cells	9
NKT cells	44
NK cells	4
Secretory cells	1949
Squamous	–
Tuft cells	13
T cells	–

#### Assessment of potential safety issues

**Figure 4:**
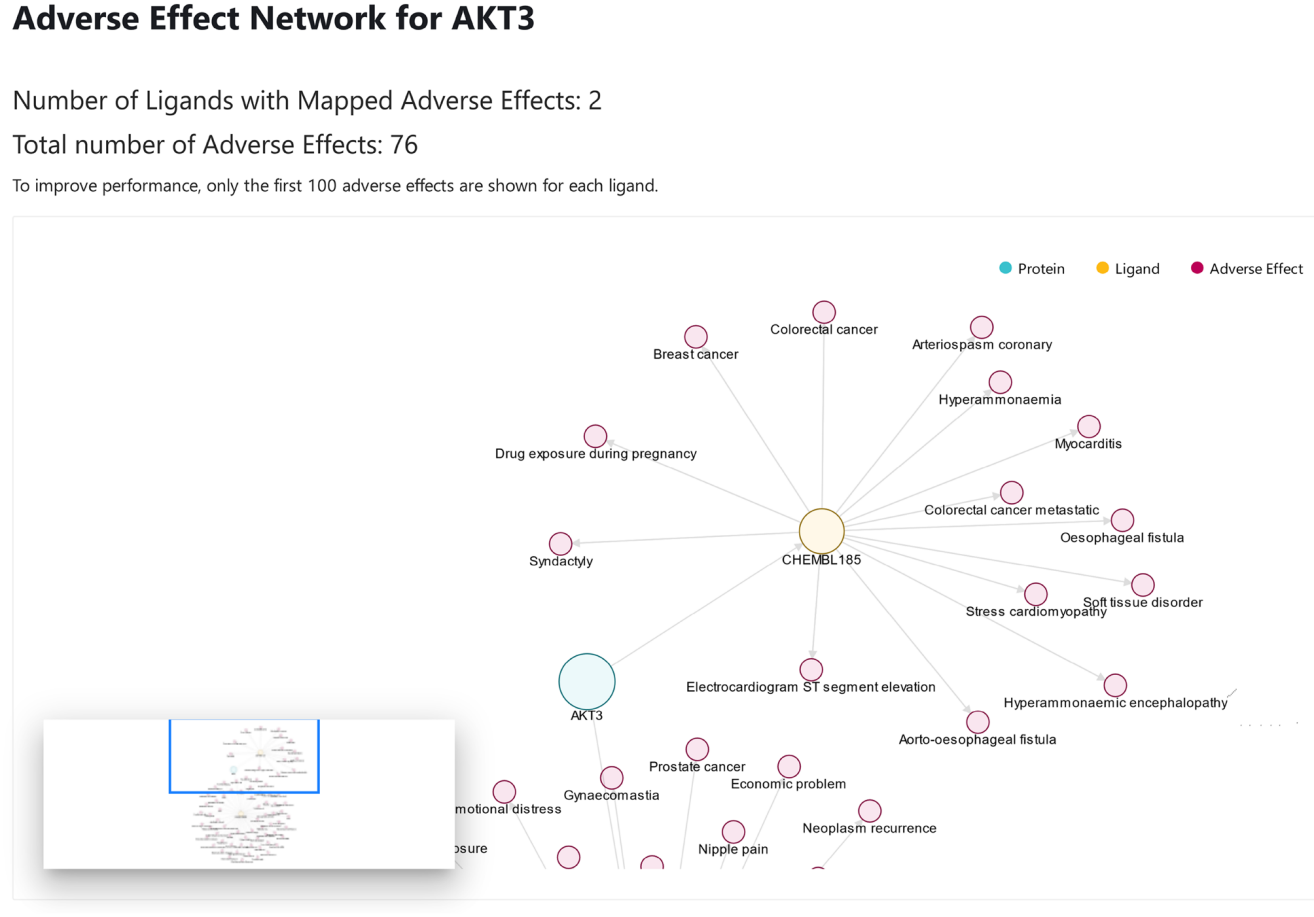
Screenshot of part of the adverse effect network for the AKT3 protein.

An important factor that must be taken into consideration with new target candidates are the adverse events which are associated with the drugs targeting these proteins. To better assess the suggested therapeutics, we mapped significant adverse effects from the NSIDES database (http://tatonettilab.org/offsides) to the extracted ChEMBL compounds. Hence, each protein can be visualized in tandem with the ligands that target it, as well as any side effects found to be associated with the linked compounds. To showcase this feature, Fig. 4 depicts the AKT3 protein as well as its associated ligands and their side effects as shown in the GuiltyTargets-COVID-19 web application.

## Discussion

Vaccinations have proven to be one of the most powerful weapons against COVID-19 despite their reduced effectiveness against the omicron variant^29^. However, even in highly developed countries such as the USA, the fraction of fully vaccinated individuals is currently still below 70%^30^. Hence, there is still an unmet need for effective and cost-efficient medications against COVID-19.

The contribution of this work is a machine learning supported web tool for (i) prioritizing novel candidate targets against COVID-19 and assessing their druggability, (ii) linking these targets to known disease mechanisms, (iii) mapping active ligands to the proteins, and (iv) pointing out the potential side effects of the suggested compounds. To our knowledge, there is currently no comparable software tool available to support such comprehensive COVID-19 drug development.

We evaluated the underlying GuiltyTargets algorithm using six bulk RNA-Seq and three single cell RNA-Seq datasets, in total spanning 17 different cell types. Based on this data, we showed that our tool not only provides a high ranking performance which is in agreement with our previous publication, but also consistently prioritizes proteins that have a clear disease association. Additionally, we demonstrated that our tool could be used to explore candidate targets which are unique to specific cell types. To facilitate the subsequent drug development process, our GuiltyTargets-COVID-19 tool provides an assessment of druggability, a network mapping of candidate targets, a mapping of active ligands from ChEMBL, and a linkage to potential side effects.

Though we were able to find shared targets among the dataset groups, there are likely additional viable targets that were not identified due to the limited amount of data we had access to at the time of writing. By increasing the number of datasets in the future for either a bulk group or a specific cell type, we can likely reveal more potential targets that are common among all of the groups analyzed, thus providing additional therapeutic routes to test. Furthermore, we found a high imbalance of compounds targeting GRK2, the only protein prioritized uniquely in secretory cells. Suggesting such a large number of compounds for testing is unhelpful, and improvements will be made to our web application to better filter the ligands mapped to the ranked candidates.

In summary, we believe that our GuiltyTargets-COVID-19 web application provides a useful contribution to the scientific community and will help facilitate future drug development against COVID-19.

## Methods

### Methodological overview

We start by explaining the overall approach implemented in GuiltyTargets-COVID-19, which consisted the following steps:

1. Differential gene expression (DGE) analysis of 6 bulk RNA-Seq and 3 single cell RNA-Seq datasets.
2. Construction of a tissue specific, genome-wide protein-protein interaction (PPI) network based on data from BioGRID^21^, IntAct^22^ and STRING^23^ as well as mapping of differentially expressed genes from the 3 single cell RNA-Seq and 6 bulk RNA-Seq datasets to their counterparts within the network.
3. Labeling of known disease associated protein based on the recently published COVID-19 pharmacome^15^.
4. Training of GuiltyTargets, a positive unlabeled machine learning algorithm for prioritizing further putative drug targets based on network representation learning^19^ within each dataset.
5. Analysis of both the overlap of highly ranked targets as well as those proteins unique to specific cell types.
6. Mapping of known ligands from the ChEMBL database^31^ to the candidates.
7. Identification of potential adverse effects for the given compounds.

**Figure 5:**
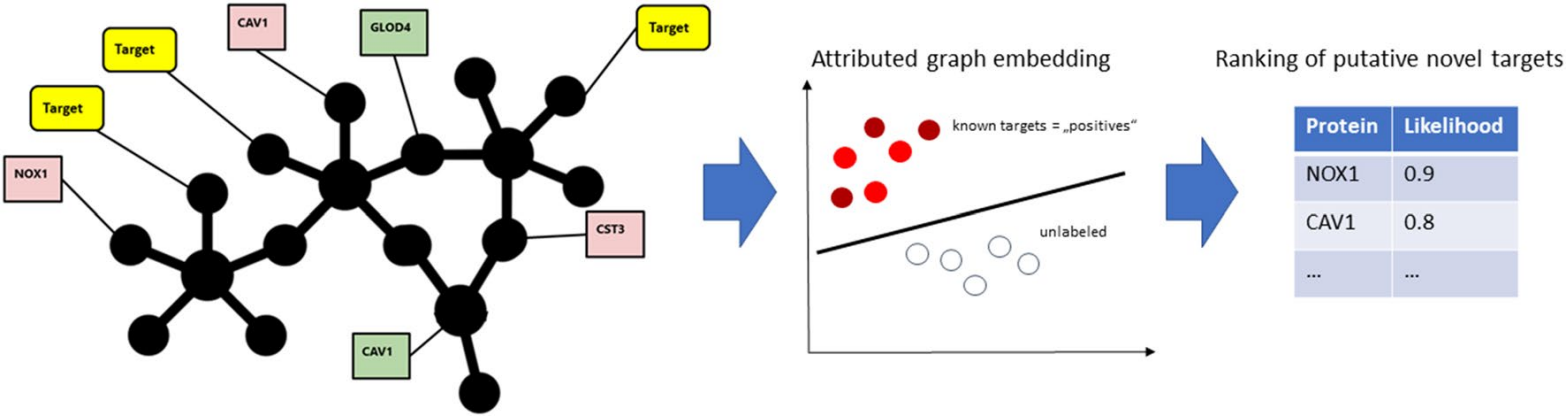
Idea behind GuiltyTargets: information about differentially expressed genes and putative COVID-19 drug targets are mapped to a constructed tissue specific PPI network. Subsequently, GuiltyTargets applies network representation learning to embed the attributed graph into an Euclidean space. This positive-unlabeled model is used to rank unlabeled proteins with respect to their likelihood of being COVID-19 drug targets.

Briefly, GuiltyTargets^19^ (Fig. 5) is a positive-unlabeled machine learning algorithm which combines a PPI network, DGE, and a list of nodes labeled as known targets for a given disease in order to rank putative novel drug targets relative to a set of existing ones. This technique quantifies the likelihood that a candidate protein could be labeled as target based on the overall similarity to existing targets (“guilt by association” principle). Given the fact that there are currently only a few approved drugs for COVID-19 (7 as of December 2022 in the European Union), we chose to employ a set of 218 potential targets involved in disease specific cellular response mechanisms that have been previously shown to be transcriptionally dysregulated in several bulk RNA-Seq datasets^15^. More details regarding GuiltyTargets can be found in Section “Machine learning based target prioritization”.

### Differential gene expression

Bulk RNA-Seq data was obtained from NCBI’s Gene Expression Omnibus (GEO) by querying the database for experiments on SARS-CoV-2 in Caco2 cells or samples directly from patients. Only those which contained a control/healthy group were included, and the raw counts were analyzed for differential gene expression (DGE) using DESeq2^32^.

Single cell RNA sequencing (scRNA-Seq) data was obtained from^33,34^, and^35^ (GSE145926). Regarding the former two, cell type specific differential gene expression results were directly provided by the respective authors. For the latter, the data was pre-processed and analyzed for cell type specific differential gene expression using the Seurat R package^36^.

### PPI network construction

To construct the PPI network, we extracted data from the BioGrid, IntAct, and STRING databases. The PPIs derived from these databases were used to create a knowledge graph consisting of protein nodes. All protein identifiers were converted to Entrez gene identifiers in order to synchronize the three resources. These databases provide confidence scores for each interaction which quantify the degree of evidence by which the interactions are supported, and were subsequently added to the network structure as edge weights. Finally, the resulting network was filtered to represent the lung proteome according to the Human Protein Atlas^37^.

### Machine learning based target prioritization

Our earlier published GuiltyTargets prioritization approach uses network representation learning to achieve a ranking of all proteins in the graph as potential drug targets based on network structure and DGE data. The DGE data used was first categorized using the following divisions: − 1 to indicate underexpressed (false discovery rate< 0.05, log2 fold change < − 1.0), 1 meaning overexpressed (false discovery rate < 0.05, log2 fold change > 1.0), and 0 or not differentially expressed. The network was subsequently annotated with the annotated DGE data as protein node features. The approach utilizes the gat2vec algorithm, which then splits the graph into two networks: one composed solely of the structural network skeleton and one bipartite graph containing only the subset of the nodes which are labeled with DGE data as well as any additional vertices representing the annotated DGE attributes themselves^38^. The gat2vec algorithm then approximates node similarity through random walks, a process in which two nodes are considered more similar the more frequently they co-occur while traversing the graph from any given starting node. Random walks are used on each of the two aforementioned networks, thus generating a structural context from the former and a attribute context from the latter. These structural and attribute contexts serve as input into a SkipGram neural network^39^ which learns representative, Euclidean-space node embeddings. Finally, the GuiltyTargets algorithm uses a l2-penalized logistic regression classifier to predict the probability of each node, or protein, in the network, of being a potential drug target. These probabilities are the foundation on which the drug target ranking is attained. As GuiltyTargets was designed specifically for prioritizing drug targets, proteins are either positively labeled as drug targets or entirely unlabeled (pseudo-negatives) rather than a more typical positive/negative labeling scheme. The positive labels were derived from the proteins defined by Schultz et al.^15^ and applied to corresponding nodes in our network. All remaining nodes (i.e. those not labeled as positive) were treated as pseudo-negatives (Fig. 5). We refer to our original publication^19^ for more details regarding GuiltyTargets and how the probabilities are estimated.

For each of the bulk RNA-Seq datasets as well as each individual cell type classified in the scRNA-Seq data, a GuiltyTargets model was trained to prioritize the proteins in the compiled lung-filtered network. After these rankings were created, the top targets were gathered and combined across all bulk RNA-Seq results as well as for all cell types from the scRNA-Seq results. In order to compare specific therapeutic routes by cell type, a list of unique prioritized targets was also generated for each dataset from the scRNA-Seq data (Table 1). These lists were generated by identifying the proteins that are unique to each cell type and not found in any other set. Targets were then mapped to any active chemical ligands found in the ChEMBL database.

### Evaluation of target prioritization performance

While previously compared our GuiltyTargets algorithm against competing methods based on multiple datasets^19^, the focus of this work is its direct application and benefits in the context of COVID-19 as presented in our web application.

**Figure 6:**
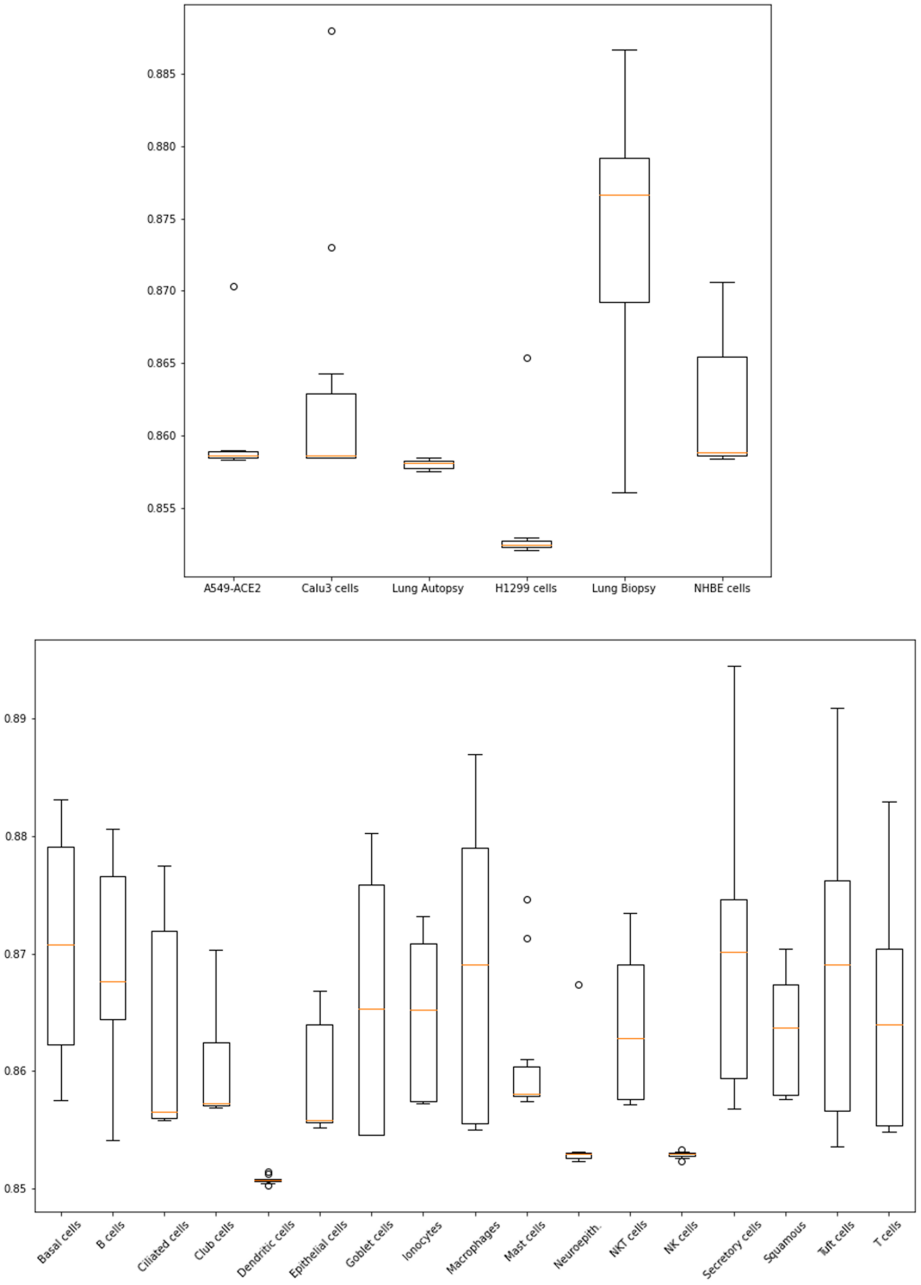
Ranking performance of GuiltyTargets measured by the AUC within a 10 times repeated, stratified 5-fold cross-validation. The boxplots show the distribution of the AUC over the 10 cross-validation repetitions. Top: performances on bulk RNA-Seq. Bottom: performances on single cell RNA-Seq.

GuiltyTargets provides a ranking of candidate proteins relative to the set of putative COVID-19 targets taken from Schultz et al.^15^, which we refer to as positives in the work presented here. Our aim was to understand the probability that GuiltyTargets would rank one of those positives higher than any unknown protein. To that end, we trained the underlying GuiltyTargets algorithm using a 10 repetition, stratified 5-fold cross-validation scheme. This ensured that each independent test set inside the repeated cross-validation procedure contained approximately the same number of known targets. We report the area under receiver operator characteristic curve (AUC) as a ranking performance measure for GuiltyTargets based on nine datasets (Fig. 6). These same results are also provided on the GuilyTargets-COVID-19 homepage and demonstrate that GuiltyTargets assigns positives with high probability a higher rank than unknown proteins, as expected. In general, AUCs observed on bulk and single cell RNA-Seq datasets are highly similar, and on a range comparable to that reported in our previous publication^19^.

### Ligand mapping

To suggest potential drugs which target the prioritized protein set, information about chemical ligands for each target was obtained from the ChEMBL API using the available Python driver. Features including the canonical Simplified Molecular-Input Line-Entry (SMILES) string, the molecule name, and the corresponding assay information regarding the discovery of each ligand were included and used for further chemical validation. Ligands were sorted by pChEMBL value, an approximate measure of potency. The pChEMBL values were calculated as the negative log of the half-maximal concentration/potency/affinity values and are therefore roughly comparable. Only ligands with a pChEML value greater than five, which corresponds to a half-maximal value of < 10 μM, were considered as potential therapeutics and subsequently mapped to the protein target.

### Automatic literature mining

To determine whether a particular protein was previously associated with COVID-19, we used SCAIView, our in-house semantic search engine that is capable of identifying co-occurring ontological terms within primary literature. Briefly, ontologies describing all currently accepted gene symbols (as defined by the HUGO Gene Nomenclature Committee) as well as COVID-19^40^ and SARS-CoV-2 were loaded into our COVID-19 SCAIView instance and a massive collection of recent publications were parsed and annotated with matching terms from these ontologies. Gene symbols that were found in the same publication as a COVID-19 or SARS-CoV-2 ontology term had their corresponding protein node labeled as being associated to the disease. The semantic search engine described here is available for public use at https://covid.scaiview.com.

## Supplementary Information

Supplementary Legends.

Supplementary Information 1.

Supplementary Information 2.

## Data Availability

The code and all data used in the analysis is available to download at https://gitlab.scai.fraunhofer.de/bruce.schultz/gtcovid. Data sources are described in the “Differential gene expression” section.
