# Supplementary material for: A machine learning method for the identification and characterization of novel COVID-19 drug targets
Source: Sci Rep. 2023 May 3;13:7159. doi: 10.1038/s41598-023-34287-5 (PMC10156718; doi:10.1038/s41598-023-34287-5)
Supplement: Supplementary file 1 — Supplementary Legends. [file 41598_2023_34287_MOESM1_ESM.docx]

**Supplementary Information**

Supplementary Data S1: **The first-degree neighboring proteins for AKT3 as described in the analyzed PPI network.** The table includes information for each protein neighbor including its Entrez identifier, HGNC symbol, and whether the protein was found to be associated with humans or the SARS-CoV-2 virus.

Supplementary Data S2: **The first-degree neighboring proteins for PIK3CA as described in the analyzed PPI network.** The table includes information for each protein neighbor including its Entrez identifier, HGNC symbol, and whether the protein was found to be associated with humans or the SARS-CoV-2 virus.
